# The Unfolding of Amyloid's True Colors

**DOI:** 10.1371/journal.pbio.0040008

**Published:** 2005-11-29

What do neurodegenerative diseases and suntans have in common? Scientists at the Scripps Research Institute have found an intriguing molecular connection. In neurodegenerative disorders such as Alzheimer and Parkinson disease, proteins aggregate into specific fibrous structures (called a cross-β sheet) to form insoluble plaques known as amyloid. Because amyloid accumulation can be highly toxic to cells and organisms, often leading to neurodegeneration, therapeutic strategies for treating such protein-conformation disorders involve targeting and reducing amyloid formation and accumulation. But in a new study in *PLoS Biology*, Douglas Fowler, Atanas Koulov, and colleagues present evidence that the amyloid structure may play a normal role in mammalian cells. Amyloid fibrils are present in melanin-producing cells in great abundance, the authors show, where they help synthesize the sunburn-fighting pigment, melanin. [Fig pbio-0040008-g001]

**Figure pbio-0040008-g001:**
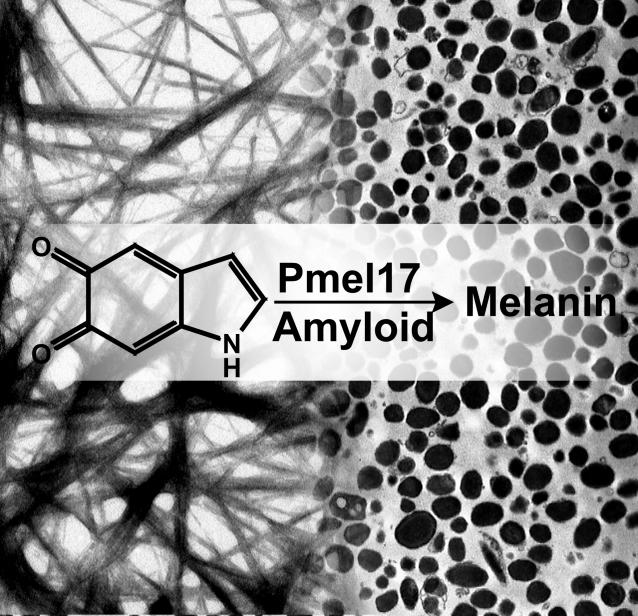
A native, nontoxic mammalian amyloid structure, generated by the protein Pmel17, helps accelerate synthesis of melanin

These pigments are synthesized in organelles called melanosomes, which reside in specialized skin cells (melanocytes) and the eyes (retinal pigment epithelium), to produce and traffic pigments for coloration, ultraviolet protection, and chemical detoxification. Melanosome biogenesis proceeds via a specialized pathway, related to the pathway producing a broad range of “housekeeping” organelles, including lysosomes, known for engulfing and cleaving, or lysing, proteins. Fowler, Koulov, and colleagues isolated melanosomes from retinal pigment epithelium taken from cows' eyes, and probed them for different protein compositions. Though the authors suspected that melanosomes might contain amyloids (based on previous reports that melanosome proteins resisted denaturation, a property of most amyloid fibers), they were surprised to find the organelle loaded with fibrillar amyloids. They visualized the amyloids primarily by using fluorescent molecules exhibiting selective binding to the characteristic amyloid cross-β sheet conformation, a fluorescent microscopy method long used by pathologists to diagnose protein-conformation disorders.

Which protein contributed to the alarming abundance of the amyloid structure in melanosomes? Several clues pointed to the glycoprotein Pmel17, a critical component of melanosome biogenesis, according to genetic and biochemical data. During melanosome biogenesis, Pmel17 lyses into two fragments, one called Mα that is sequestered into a membrane-bound compartment of the melanosome and another that is degraded. After confirming that the amyloids were comprised of Mα fibers, the authors tried to make Mα fragments fold into amyloid in a test tube. They showed that a purified, nonaggregated Mα (which they called recombinant rMα) folds into amyloids remarkably quickly. When Fowler et al. compared the rate at which rMα forms amyloids to that of other well-known amyloids—Aβ and α-synuclein, which are implicated in Alzheimer and Parkinson disease, respectively—they found that rMα amyloid production was at least four orders of magnitude faster. The authors offer the intriguing hypothesis that by rapidly folding into the amyloid cross-β sheet structure, Mα avoids generating the toxic intermediates that are very common in pathogenic amyloid formation.

Finally, Fowler et al. satisfy a burning question: are Mα amyloid fibers serving a function in melanin synthesis? After reconstituting components of the melanin biosynthethic pathway in vitro, they showed that adding rMα results in a 2-fold increase in melanin production (as does adding other amyloids like Aβ and α-synuclein). Perhaps more importantly, the Mα amyloid fibrils bind and orient the highly reactive organic melanin precursors, mitigating the cellular toxicity observed when Mα amyloid production is halted by mutation.

The authors also raise the intriguing idea that, given the propensity for many proteins to form amyloid fibrils, this conformation may be another physiologically important protein fold found in cells. To differentiate the biologically functional amyloid from pathogenic amyloids, the authors suggest using the term “amyloidin.” Although the common involvement of amyloids between melanin synthesis and protein conformation disorders is most surprising, future research into the differences between amyloid formation in these processes may hold the key for understanding diseases including Huntington, Parkinson, and Alzheimer disease. Because melanosome biogenesis is a tightly regulated process, a deeper understanding of the mechanisms that allow the Pmel17 Mα fragment to avoid the toxic stage of amyloid formation could provide considerable insight into which aspects are missing when proteins misfold. —*Jami Milton Dantzker*

